# The role of Indonesian patients’ health behaviors in delaying the diagnosis of nasopharyngeal carcinoma

**DOI:** 10.1186/s12889-017-4429-y

**Published:** 2017-05-25

**Authors:** R . Fles, A. C. R. K. Bos, Supriyati, D. Rachmawati, E. Waliyanti, I. B. Tan, S. M. Haryana, M. K. Schmidt, F. S. T. Dewi

**Affiliations:** 1 Department of Head and Neck Surgery and Oncology, The Netherlands Cancer Institute, Plesmanlaan 121, 1066CX Amsterdam, The Netherlands; 2 Department of Research, Netherlands Comprehensive Cancer Organization, Utrecht, The Netherlands; 3 Department of Health Behavior, Environment and Social Medicine, Faculty of medicine, Universitas Gadjah Mada, Yogyakarta, Indonesia; 4 Department of Otorhinolaryngology, Head and Neck Surgery, GROW - School for Oncology & Developmental Biology, Maastricht University Medical Center, Maastricht, The Netherlands; 5 Department of Otorhinolaryngology, Dr. Sardjito General Hospital - Faculty of Medicine, Universitas Gadjah Mada, Yogyakarta, Indonesia; 6 Department of Histology, Cell and Tumor Biology, Faculty of Medicine, Universitas Gadjah Mada, Yogyakarta, Indonesia; 7 Division of Psychosocial Research and Epidemiology, Netherlands Cancer Institute, Amsterdam, The Netherlands; 8 Division of Molecular Pathology, Netherlands Cancer Institute, Amsterdam, The Netherlands

**Keywords:** Delay, Health behavior, Interview, Nasopharyngeal carcinoma, Qualitative research

## Abstract

**Background:**

With an estimated 13,000 newly diagnosed patients per year, nasopharyngeal carcinoma (NPC) is one of the most common types of cancer in males in Indonesia. Moreover, most patients are diagnosed at an advanced stage of the disease. This study aimed to explore the health behaviors of patients diagnosed with NPC and the possible causes of patient delay in NPC diagnosis.

**Methods:**

A qualitative research method was used to gain better insight into patient behaviors. Twelve patients were interviewed using semi-structured interview guidelines. All interviews were recorded, transcribed verbatim and analyzed according to a standard content analysis framework.

**Results:**

Most patients had limited knowledge regarding NPC and its causes. Fifty percent of the patients had a delay of six months from the onset of symptoms to diagnosis. The main reason for this delay was the lack of awareness among the patients, which was influenced by their environment, economic status, family, culture, and religion. The perceived barriers to seeking medical help included direct non-medical costs not covered by health insurance, complex and time-consuming insurance and referral systems, and negative experiences in the past. Health insurance did motivate people to seek medical help.

**Conclusion:**

This study provides additional insight into patients’ motivations to delay seeking medical help and can facilitate the design of NPC education programs. To improve awareness of the abovementioned causes for delay, community-based education programs are highly warranted and should focus on the recognition of NPC symptoms and possible solutions to overcome the main barriers at an earlier disease stage.

## Background

Cancer mortality is rising in middle- and low-income countries, while increased numbers of cancer survivors can be found in high-income countries [1]. Moreover, a delayed diagnosis of cancer is more often found in middle- and low-income countries due to the limited availability and accessibility of health care. Patients’ ability and willingness to undertake action, which depends on their social-economic status, attitude, financial situation, culture, and religion, also play a role [2–5]. In addition, patients are often embarrassed to discuss abnormalities [4, 6] or lack trust in medical consultation [7].

Nasopharyngeal carcinoma (NPC), with over 13,000 newly diagnosed patients per year, is one of the most frequently encountered types of cancer in Indonesia, especially in males, and is associated with high mortality [8]. Early symptoms of NPC are nonspecific and mimic a normal upper airway infection, making it a difficult disease to recognize for general practitioners working in primary health care centers. A previous study conducted in Yogyakarta, Indonesia, showed that most patients were diagnosed with an advanced stage of NPC and had a median overall survival of less than 2 years [9].

In Javanese culture, health is seen as a requirement to complete daily activities, and as long as the Javanese are not hindered in their daily activities, they consider themselves to be healthy [5]. Javanese men are the breadwinners of the family and the decision-makers after consulting family members and considering their partner’s opinion. Women, on the other hand, take care of the health of the family [5]. The ability of patients in Indonesia to seek medical help often depends on the role of the family [10, 11]. The importance of the family is reflected in the following proverb: ‘mangan ora mangan waton kumpul’ (even if there is no food to eat, being together is the most important thing) [12].

Delay in diagnosis can occur in the form of patient delay, doctor delay, or system delay. Poor health literacy influences patient behavior and often plays a role in delayed diagnosis and poor treatment outcomes [13, 14]. A systematic review and an integrative review investigated patient delay in patients with head and neck cancer [7] and oral cancer [15]. However, the studies described by Goy et al. focused on the relation between diagnostic delay and stage at diagnosis and did not examine possible explanations for this delay [7]. Additionally, NPC is often excluded in these studies, which mainly report patient delay in high-income countries. To the best of our knowledge, qualitative research exploring health behaviors in patients diagnosed with NPC in Indonesia has not been reported.

Therefore, the aim of this qualitative study was to explore and understand health behaviors and the possible causes of patient delay in the diagnosis of NPC. By better understanding this context and environment, we believe that community-based interventions focused on minimizing patient delay in NPC diagnosis and better treatment outcomes can be efficient.

## Methods

### Participants and procedure

Between March 2014 and June 2014, patients newly diagnosed with NPC at the Ear, Nose and Throat Department of Dr. Sardjito Hospital were recruited. The inclusion criteria were age older than 18 years, having histopathologically confirmed NPC, currently waiting for radiotherapy, and understanding and speaking Bahasa Indonesian or Javanese. Patients who had a psychiatric treatment history or were too ill to complete the interview were excluded (one interviewed patient was excluded in retrospect). The Medical and Health Research Ethics Committee of Gadjah Mada University, Yogyakarta, Indonesia approved the study.

### Data collection

The in-depth, semi-structured interview design was based on the health belief model (HBM). The failure of individuals to enter interventions that prevent diseases is well-explained by the HBM [16]. The HBM predicts the behavior of individuals based on two variables: the values of the individual and the perception of the individual that an action will result in achieving a particular goal [17]. The HBM consists of the following categories: perceived susceptibility, perceived severity, perceived benefits, perceived barriers, and cues to action [18]. In this study, the HBM may reflect the perception of NPC patients of an NPC diagnosis and the delay in diagnosis of NPC patients. Therefore, this model was chosen to form a conceptual model, which was used as the basis for the development of the sub-questions in our study. Based on the HBM categories, sub-questions and main topics were discussed (AB, RF, FD, and SS) and adapted if needed. Pathway to diagnosis was defined as date from the onset of symptoms until histological proven date of diagnosis.

All eligible patients received a short oral explanation of the study while visiting the outpatient clinic. The anonymity of the respondents was guaranteed, and all patients provided informed consent. Each interview lasted approximately 60 min and was conducted in Bahasa Indonesian or Javanese. To ensure that the patients were at ease, a trained interviewer conducted the interviews at the patients’ homes. Participants were asked about their demographic characteristics, including age, religion, insurance status, level of education, and employment status. These questions were followed by an in-depth, semi-structured interview on topics related to NPC.

Each interview was recorded, transcribed verbatim, translated into English, subsequently reviewed by a bilingual team member (AB), and coded using content analysis using MaxQDA11. Following the emergent design, the coding scheme was jointly developed by FD and SS to decide on new probing questions and the saturation of answers according to theoretical saturation. Saturation was reached after 12 interviews. Participant inclusion ended when no new concepts arose from the last two interviews. The key themes were pre-determined (i.e., perceived susceptibility and severity, perceived benefits and barriers, cues to action, and the influence of culture and religion). All codes derived from the interviews were compiled into categories and merged into the main themes using MAXQDA11. This process was conducted in two steps: after six interviews and at the end of data collection. Inter-coder agreement of 85% was measured over a sample of interviews, and differences were discussed until a consensus was reached. Final coding and categorization were performed by two researchers (RF and AB). Trustworthiness was achieved through different cultural backgrounds within our research team. Having team members with different cultural background ensured that culturally embedded barriers were revealed. Additionally, by interviewing patients in their homes, patients were more likely to be at ease, resulting in less social desirability bias.

## Results

### Characteristics of the participants

This study was based on data from 12 interviewed NPC patients. For two patients, a relative of the patient participated during the interview because the patient insisted on it. Two-thirds of the patients were male, their mean age was 44 years, and all participants were Muslim and of Javanese origin (Table 1). Six participants had a low educational level. Most patients had insurance for (borderline) lower income individuals. All patients had an advanced stage of disease, and two of them already had distant metastasis at diagnosis. For ten patients, the delay in diagnosis after the first symptoms was more than three months. Most patients visited multiple health care institutions before their appointment at the Ear, Nose and Throat Department of Dr. Sardjito Hospital. One patient even visited eleven different health care institutions.

**Table 1:** Characteristics of the participants

Patient characteristic	Category	n	(%)
Gender	Male	8	66.7
	Female	4	33.3
Age	<35	3	25.0
	35–45	3	25.0
	45–55	4	33.3
	>55	2	16.7
Ethnicity	Javanese	12	100.0
	Other	0	0.0
Religion	Islam	12	100.0
	Other	0	0.0
Level of education	No school	1	8.3
	Did not complete primary school	0	0.0
	Graduated primary school	5	41.7
	Graduated junior high	3	25.0
	Graduated high school	2	16.7
	Graduated from university	1	8.3
Occupation	Housewife	3	25.0
	Entrepreneur	3	25.0
	Civil servant/Police/Army	1	8.3
	Farmer/Fisherman/Labor	4	33.3
	Student	1	8.3
Marital status	Not married	2	16.7
	Divorced	0	0.0
	Widow	1	8.3
	Married	9	75.0
Types of insurance	Lower income	10	83.3
	Other	2	16.7
Stage disease	III	1	8.3
	IVA	2	16.7
	IVB	7	58.3
	IVC	2	16.7
Time from first symptoms to diagnosis in months	<3	2	16.7
	3–6>	4	33.4
	6–9>	5	41.7
	≥9	1	8.3

### Perceived susceptibility and severity (quotes 1–9; Table 2)

Most patients (*n* = 10) had never heard of NPC before their diagnosis or said they had limited, basic knowledge of NPC and its causes (*n* = 8). When patients were asked about the perceived causes for their illness, they often did not know. However, having cancer frightened the patients, and they wanted to start curative treatment. In addition, they stated that they only received oral information from the doctor directly after diagnosis, and no written information was available. Some patients gathered information on the symptoms and the risk factors through their doctor, family, friends, or the internet. However, not all patients were interested in obtaining knowledge about the disease. Several patients changed their lifestyle (e.g., quit smoking), family lifestyle, and/or food consumption (e.g., not using flavor enhancers like MSG and minimizing the consumption of salty fish).

**Table 2:** quotes of the participants

nr	patient	Quote
		Perceived susceptibility and severity
1	7	*“I never heard of NPC before […] even my doctor was surprised […] I was just shocked”.*
2	2	*Statement by daughter of this patient:* “*Personally, I have no idea what causes the disease…before, my father ever had a ruptured blood vessel.”*
3	11	*“I’m afraid of cancer…..I am afraid that I would die.”*
4	12	*“even though I have this illness but I don’t consider myself to have this illness, just like normal.*
5	8	*“I totally changed my lifestyle. I totally quit smoking and I also eat vegetable, it feels good”.*
6	4	*“if I would not have waited, but went straight to the dr. Sardjito hospital, I would still be like this”*
7	6	*“At first, I had a headache, I was dizzy and all the bones in my left side from the bottom are painful; then my son told me to go check it in the hospital”.*
8	5	*“I had a small mass in my neck, more than one year ago, then I had a biopsy. But the mass came back five months ago. During the time I regularly had blood in my sputum and a headache, but I did not think that was serious.”*
9	9	*“I had a ringing sound in one of my ears. After a while I went to the doctor, but he said that I was fine, so I thought it was just flu. But then I had a terrible headache and I had a mass in my neck. The mass really felt abnormal, so I went to the doctor again.”*
		Perceived benefits and barriers
10	9	*“According to the theory I would get cured […] the chemo would make the illness less and the radiotherapy would eliminate it”.*
11	8	*“I was diagnosed with a bronchitis […] after several months the lumps in my neck appeared”.*
12	1	*“I got a tonsil operation, I thought I would be cured, but I still had a headache […] I went to eleven different doctors before I got to the Ear Nose Throat doctor […] now we only have to wait for the chemotherapy”.*
13	11	*“For registration I had to wait for one to two hours…… after I got the schedule for radiotherapy, I wanted the internist to tell about the schedule…. I had to queue from the beginning again”*
14	9	*“I would like to pay no matter what, my son told me not to use BPJS they would treat us like less important”.*
15	3	*“There was a patient from Banyumas; she had a blackened face, I’m only afraid I will get that too when I start radiotherapy”.*
		Cues to action
16	8	*“My suggestion is there should be posters in the street so they know about the danger of smoking […] I often see young kids on the street smoking, only thing I can do is tell them about the risk of getting cancer, because I already have the experience”.*
17	7	*“I went for 8 times to dr. Sardjito hospital […] until all papers were complete. I was there until I fainted […] we had to go there and there”.*
18	8	*“The queue for the registration is very long […] this is an obstacle; this should be easier”.*
19	3	*“About the costs, it is hard […] but the cost to go there by bus is too much […] For the treatment we now use BPJS, the insurance before only gave us a discount, but now we do not have to pay for the treatment”.*
		Culture and religion
20	10	*“My health was decreasing, so my family and kids quickly decided to get medical treatment […] We would do everything to be able to pay for it […] it was money from the family”.*
21	2	*“We didn’t tell my father about his disease… we are afraid that he will get stressed [..] He knows that he is sick”.*
22	2	*“I don’t know the names of all the herbs; knowledge is inherited from previous generations, my grandparents […] I’m using alternative treatment because I just want to be cured. Besides that, it is more affordable”.*
23	10	*I do want to know more clearly, well, because I am only a patient so I keep quiet […] maybe if I asked something, the doctor would be offended.*
24	7	*“If God allows I will be cured”*
25	10	*“I am not afraid, the most important thing is that I totally surrender to have this illness, hope God will take it away”.*

Most respondents waited several months before seeking medical help, and some did not realize that delaying the diagnosis could worsen the disease. The median delay before seeking medical help was 5.5 months (range 1–12), during which time the respondents observed that their symptoms became more severe. The patients did not recognize the first symptoms of NPC; these symptoms were often considered to be harmless because they did not hinder the patients in their daily activities. The symptoms described by the patients as mild included a ringing sound in the ear, headache, runny nose, nose bleeds, double vision, and symptoms of the common flu. Some patients described the pain or the mass in their neck as mild and not severe, although a neck mass is one of the symptoms of more advanced stages of the disease. When symptoms like a headache and enlarged neck lymph nodes did not disappear over time, it was a trigger to seek medical help. One patient, who described severe pain in the bones, was later diagnosed with distant metastasis.

### Perceived benefits and barriers (quotes 10–15; Table 2)

Most patients were unaware of the severity of their disease and mainly expected to get rid of their symptoms as they would a headache. The primary motivation for patients to seek medical help was to get healthy again.

Although many patients mentioned positive experiences with the health care system, all patients shared negative experiences with health care services resulting in a barrier to seeking medical help. Patients often encountered physicians who were unaware of the disease, resulting in misdiagnosis and time-consuming referrals, followed by long queues due to limited treatment capacity. In addition, while waiting, patients often encountered more severe cancer patients, which frightened the patients even more. All of these experiences ultimately formed barriers for the patients to continue visiting health care providers, and they often looked for alternatives.

Medical costs often resulted in patients postponing medical treatment. Although insurance covered most of the costs, patients only started applying for this insurance once the need for medical help was inevitable. Applying for insurance is time consuming, and the insurance does not cover costs like transportation. Frequently, patients had to travel long distances and had to rent a car. In addition, not all patients wanted to use the insurance because they felt that the treatment and service was different when they paid themselves.

Lack of awareness and fear of the side effects of the treatment made patients reluctant to be diagnosed or even start treatment. Experiences in their surroundings (e.g., family or friends) of chemotherapy and radiotherapy scared the patients.

### Cues to action (quotes 16–19; Table 2)

Patients stated that if they would have been more aware of the severity of the disease and the need for treatment from the onset of the first symptoms, they would have sought medical help at an earlier stage. Some patients suggested creating community-based awareness using printed media, such as folders and flyers about the symptoms and risk factors of NPC and how changes in lifestyle may minimize the risk of developing NPC. In addition, improving the referral system and queuing system for registration would help patients to overcome these barriers and seek medical help at an earlier time point.

Although transportation costs are still not covered by health insurance and can be seen as a barrier to seeking medical help, the introduction of health insurance did motivate patients to take action. In the past, treatment was unaffordable for some patients, but the introduction of an insurance system has changed this situation.

### Culture and religion (quotes 20–25; Table 2)

**Fig. 1:**
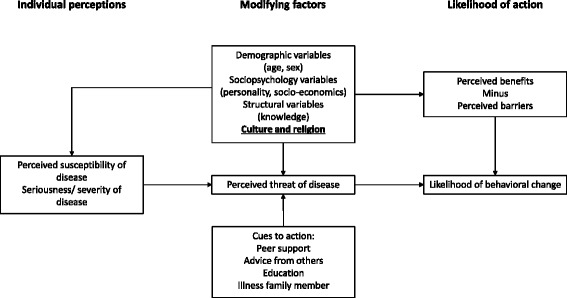
Health Believe Model according to Hochbaum, Rosenstock and Kegels [15] with of culture and religion added as modifying factors by the authors

The participants were strongly bound to Javanese culture and religion, and for that reason, these two aspects were added to the HBM (Fig. 1). Religion and the support of family members to get diagnosed or undergo treatment play a large role in Indonesian culture. In addition, family support is needed to cover possible extra costs. With support from family and friends, most patients start with traditional, complementary, and alternative medicine (TCAM) before seeking medical treatment. The main motivation for using TCAM was to minimize symptoms, as patients were unaware of the underlying disease. Patients often stated that TCAM was easier to obtain and more affordable. However, they also stated that a limited reduction in symptoms was experienced.

The one-way style of communication of health care providers instead of a partnership communication style, as is present in Indonesian culture, often creates a barrier for the patient. The dominant role of the doctor hinders the patients from asking questions, and patients are too modest to ask for more information or to question the treatment options.

Moreover, all patients in this study were Muslim. Their strong beliefs in God ensured that they accepted the consequences of the disease, resulting in more passive health behaviors.

## Discussion

This study addressed the health behaviors of Indonesian patients newly diagnosed with NPC before they received medical treatment. The following factors were found to influence the patients’ health behaviors: environment, economic status, the complex and time-consuming insurance and referral system, lack of awareness, negative experience in the past, family, culture, and religion. Culture-embedded barriers are often difficult to distinguish from one’s own culture. It was a great asset that our research team was multicultural and the phenomena could be examined from different angles. In this way, culturally based barriers were discovered. By adding cultural and religious aspects to the HBM, the model became more representative of the different factors associated with the delay in diagnosis of people experiencing the first symptoms of NPC.

In a Malaysian study by Prasad et al., patients with NPC presented themselves to a doctor within a reasonable time, but for one-third of the patients, it took more than six months to receive a diagnosis [19]. In our study, we specifically looked at the time between the onset of symptoms and the diagnosis instead of the time until the first visit to a doctor. We investigated this time period because all patients except one visited multiple health care providers before being diagnosed at the ENT department at Dr. Sardjito Hospital. We found that for 50 % of the patients, it took more than six months to receive a diagnosis.

Although NPC has a high incidence in Indonesia, participants were not aware of the causes or severity of the disease. This lack of knowledge regarding NPC seemed to influence the stage at diagnosis and the patients’ perceptions of their susceptibly to and the severity of the disease. These findings confirmed that knowledge is a key predictor in patient delay, which is in concordance with other studies of patient delay for breast cancer diagnosis in developing countries [20–23]. Most participants reported the first symptoms of NPC as harmless and compared them to symptoms of the common flu. As a result, all patients were diagnosed with an advanced stage of the disease accompanied by enlarged lymph nodes in the neck. The misinterpretation of cancer symptoms is a problem that has been reported elsewhere [24, 25]. Additionally, patients were not aware of the consequences of a delay in diagnosis.

The costs of treatment, if not covered by insurance, and the cost of transportation to the hospital caused major treatment-related delays. This finding is in line with other studies that examined treatment outcomes for childhood cancer in Indonesia [26, 27]. Although insurance stimulated patients to seek medical help, patients still struggled with direct non-medical costs.

Indonesian culture often shows hierarchical respect towards people with a higher social status and the elderly, and maintaining harmony between people is seen as important [28]. This respect towards medical staff often leads to unmet information needs and low satisfaction with the provided information. This low satisfaction may lead to unfavorable outcomes, reduced understanding of the illness, and lower health-related quality of life [29–32].

### Limitations

Because theoretical saturation was achieved after 12 interviews, no new patients were included, resulting in a relatively small study population. However, the sample size was adequate because the research scope was narrow, and the study population was homogenous [33]. Thus, one can argue that the sample size was large enough to represent patients diagnosed with NPC in Indonesia. Additionally, we should note that there might have been some recall bias. All patients were diagnosed with an advanced stage of the disease. Although their condition was good enough to participate, one can imagine that it may have been difficult for the patients to remember their onset of symptoms and exact referral path. Often, the recollection of patients was based on a public holiday or other event. However, the level of detail the patients could recall suggested the validity of the interviews. However, for that reason, in the pathway to diagnosis we did not make any further distinguish between the appraisal and help-seeking intervals suggested by others [34–36]. In the period from the onset of symptoms and diagnosis also other factors influences the delay e.g. the doctor’s delay and primary care and diagnostic interval [37].

The HBM is a theory used to describe or predict behavior at an individual level. This theory illustrates one’s willingness to act on perceived threats and possible outcome of the actions, based on logical reasoning. We used HBM to understand why an individual suffering from the symptoms of NPC, delays seeking help and still considers the illness as a mild condition with low benefits for early treatment and faces high barriers to seek help. At a later stage in the pathway to diagnosis, the HBM theory is no longer applicable, as the disease has been perceived as life-threatening, not only requiring immediate treatment, but also, requiring more actions from the individual beyond his abilities. Therefore, culture and religion will play a more important role in individuals’ way to handle the problem.

## Conclusions

Half of the patients with NPC were diagnosed more than six months after the onset of symptoms, and all patients had an advanced stage of the disease. The main reason for this delay was the lack of awareness of the patients, which was influenced by their environment, economic status, family, culture, and religion. The complex and time-consuming insurance and referral system, negative experiences in the past and direct non-medical costs not covered by health insurance were the greatest barriers to seeking treatment. Improved and varied information services in combination with more effective referrals are important goals to minimize the delay in diagnosis for NPC patients. Community-based education is needed and should focus on the recognition of NPC symptoms and possible solutions to overcome the main barriers at an earlier stage of disease.

## Abbreviations

NPC: Nasopharyngeal carcinoma
TCAM: Traditional Complementary and Alternative Medicine
